# Regional differences in recommended cancer treatment for the elderly

**DOI:** 10.1186/s12913-016-1534-z

**Published:** 2016-07-15

**Authors:** Vivian Ho, Meei-Hsiang Ku-Goto, Hui Zhao, Karen E. Hoffman, Benjamin D. Smith, Sharon H. Giordano

**Affiliations:** Rice University’s Baker Institute for Public Policy, Rice University, 6100 Main Street MS-40, Houston, TX 77005 USA; Department of Economics, Rice University, 6100 Main Street MS-40, Houston, TX 77005 USA; Department of Medicine, Baylor College of Medicine, One Baylor Plaza, Houston, TX 77030 USA; The University of Texas MD Anderson Cancer Center, Houston, TX 77030 USA; Department of Health Services Research, The University of Texas MD Anderson Cancer Center, 1400 Pressler Street, Unit 1444, Houston, TX 77030 USA

**Keywords:** Treatment variation, Specialists, Guidelines, Colorectal cancer, Pancreatic resection, Prostate cancer

## Abstract

**Background:**

Little is known about regional variation in cancer treatment and its determinants. We compare rates of adherence to treatment guidelines for elderly patients across Texas and whether local specialist supply is an important determinant of treatment variation.

**Methods:**

Previous literature reviewed indicated 7 recommended courses of treatment for colorectal, pancreatic, and prostate cancer. We analyzed Texas Cancer Registry data linked with Medicare claims for the years 2004 to 2007 to study patients with these cancers. We tested for unadjusted and adjusted differences in treatment rates across 22 hospital referral regions (HRR). We tested whether variation in the local supply of specialists treating each cancer was an important determinant of treatment.

**Results:**

We found significant differences in adjusted treatment rates across regions. For removal and examination of 12+ lymph nodes with colon cancer resection, 13 of 22 HRRs had rates significantly different from the median region. For adjuvant chemotherapy for regional colon cancer, five HRRs significantly differed from the median. For prostate cancer treatment with a favorable diagnosis, nine HRRs differed from the median HRR. Of the 7 treatments, only the local availability of surgeons was an important determinant for excision of lymph nodes for colon cancer patients.

**Conclusions:**

There are significant variations across Texas for seven recommended cancer treatments. No one region has consistently higher or lower treatments than other regions, and local specialist supply is not an important predictor of treatment. Different factors may be determining regional variation in treatment rates across cancer types and treatment options.

**Electronic supplementary material:**

The online version of this article (doi:10.1186/s12913-016-1534-z) contains supplementary material, which is available to authorized users.

## Background

Overall cancer mortality has declined in the U.S. over the past several decades, and most site-specific cancers have also experienced decreased mortality rates [1]. Advances in screening and treatment have contributed to reduced death rates from cancer over time. However, as medical technology for treating cancer becomes more complex and the options for treatment become more diverse, it also becomes more challenging to guarantee that all patients are receiving high quality treatment, given their diagnosis.

This study compares rates of high quality treatment for patients aged 65+ across regions of Texas. Several studies have identified regional variation in treatment for individual cancer types [2–10]. Past studies comparing risk adjusted Medicare spending across regions give the impression that some areas tend to treat patients of all types more aggressively than others [11–13]. However, no study has simultaneously compared treatment rates for multiple cancer therapies to determine whether certain areas tend to treat patients at a higher or lower rate relative to other regions.

In addition, not all past studies have compared treatment relative to recommended guidelines for care. Physicians can obtain guidelines for cancer care from the National Comprehensive Cancer Network Guidelines, the American Society of Clinical Oncology Practice Guidelines, or from peer-reviewed manuscripts. Previous studies have identified low rates of appropriate treatment in the U.S. for a variety of cancer illnesses [1, 2, 14–17]. Factors that have been associated with variation in guideline compliance include age, comorbidities, insurance type, institution, and region [14, 17]. We compare actual treatment to recommended guidelines for seven treatments covering three site-specific cancers across Texas.

Texas is a particularly useful state in which to examine regional variation, because it is so large and diverse demographically and geographically. It is the second most populous U.S. state, with 27 million people in 2014. An estimated 113,630 new cancer cases, or 7 % of all U.S. occurrences are predicted for Texas for 2015 [18]. The state has several urban and rural areas and an ethnically diverse population. Some regions have access to large teaching hospitals, while many do not. We have access to Texas Cancer Registry data linked to Medicare claims files, which enables us to reliably determine cancer stage at diagnosis and follow the course of treatment for older patients over time and across multiple providers.

Some studies have found that the local supply of physicians is a significant determinant of survival or treatment rates for cancer [19–25]. Less research has been conducted comparing the supply of specialists who treat cancer patients to regional variations in treatment, [6, 26] although one study found that the local supply of medical specialists and acute care hospital capacity explains 41 % of variation in end-of-life health care spending across U.S. hospital referral regions [11]. We test whether the supply of specialists available to treat each cancer is a significant determinant of treatment rates for the elderly.

## Methods

### Treatments

We chose to study treatments for cancers in which we had prior research experience and were therefore most familiar with recommended guidelines. Previous literature was reviewed to identify 7 recommended courses of treatment for colorectal, pancreatic, and prostate cancer that could be readily measured with claims data. These recommended courses include 1) removal and pathological examination of at least 12 lymph nodes during resected colon cancer, 2) adjuvant chemotherapy within 4 months of diagnosis for patients under age 80 with regional colon cancer, 3) radiation therapy within 6 months of diagnosis for patients under the age of 80 with regional spread of rectal cancer who received surgical resection, and 4) postoperative adjuvant chemotherapy for patients under age 80 years with regional rectal cancer [27]. Relatively low rates of adherence to these recommended treatments have been documented in the U.S. previously [28]. 5) Pancreatic resection is recommended for patients with localized disease, or for patients with regional pancreatic cancer that is not locally advanced [29]. Low rates of pancreatic resection have also been documented in the U.S. [15]. All of these treatments were recommended courses of care during the study’s sample period.

Appropriate treatment for men with non-metastatic prostate cancer depends on disease characteristics; therefore, we distinguish between cancers with a “favorable” versus an “unfavorable” prognosis. Patients with non-metastatic prostate cancer can be treated with external beam radiation therapy, brachytherapy, prostatectomy, cryosurgery, androgen deprivation therapy [16]. However, there is no compelling data from published randomized trials that men age 65 and older with relatively low risk prostate cancer derive a survival or quality of life benefit from cancer-directed treatment [30, 31]. In fact, randomized trials demonstrate that in older men with low-risk prostate cancer, observation yields similar survival and decreased morbidity compared with up-front treatment [31, 32]. Most men with a diagnosis of low-risk prostate cancer in the United States received up-front treatment with prostatectomy or radiotherapy and are thus exposed to the risk of treatment-induced urinary dysfunction, rectal bleeding, and impotence. We define appropriate treatments for prostate cancer as 6) receipt of cancer-directed treatment within one year of diagnosis for those patients with an unfavorable prognosis, and 7) no cancer-directed treatment within one year (observation) for patients with a favorable prognosis. Relatively high rates of cancer-directed treatment have been documented for prostate cancer patients with both favorable and unfavorable prognoses [16].

### Data

We analyzed data from the Texas Cancer Registry-Medicare linked database. Medicare provides health insurance for U.S. residents who are 65 or older. The linkage was performed under the guidance of TCR, the National Cancer Institute, and the Center for Medicare and Medicaid Services. The data used in this study includes patients diagnosed with the study cancers between 2004 and 2007 and their Medicare claims through 2008. Patients in the sample met the following criteria: diagnosed and reside in Texas, age 65 years or older, first diagnosed in 2004 to 2007 and not at time of death, first primary cancer and no second primary cancer within 12 months, histology confirmation, continuously enrolled in Medicare Parts A and B pre- and post-diagnosis, and not a member of an HMO 12 months pre- and post-diagnosis. Analysis was limited to appropriate subpopulations when recommended courses of treatment designated specific cancer stages for care. Only men were included in the analysis of prostate cancer treatment.

Patient-level variables evaluated included gender, age at diagnosis, race/origin, Charlson comorbidity score using a NCI defined algorithm, [33] tumor size, cancer stage, and year of diagnosis. Age was categorized into the intervals 65–70, 70–75, 76–80, and 81+. Analysis for colon and rectal treatments was limited to patients 80 and under, based on treatment guidelines. We also controlled for certain census tract-level indicators of patient socioeconomic status. Median income in the census tract of residence from the 2000 U.S. Census was included in the TCR-Medicare linked database. Median incomes were classified into quartiles ($ <31,000; $31,000–< $39,000; $39,000–<$53,000; and $53,000+). These variables included urban/rural location, percent of individuals who do not speak English, percent of individuals who have completed at least some college, and median income. We determined the hospital referral region (HRR) that each patient resided in. HRRs represent regional health care markets for tertiary care [34]. The U.S. is divided into 306 HRRs, and 22 are in Texas.

The TCR data and Medicare billing claims were used to determine the treatments within the specific time frame of cancer diagnosis; the methods to identify the treatments have been previously described in the literature [16, 28, 29]. Patients were identified from the TCR Patient Entitlement and Diagnosis Summary file using major site groups that SEER has defined based on the primary site and ICD-O-3 morphology. The TCR contained the cancer disease stage at diagnosis for each patient.

We also classified the prostate cancer patients as favorable risk (stage T1 or T2 tumor and low histologic grade) versus unfavorable risk (T3 or T4 tumor or intermediate/ high histologic grade) [16]. PSA information is not currently captured by TCR and so could not be incorporated into patient risk stratification.

We counted the number of specialists per 1,000 Medicare elderly patients who had at least one Medicare claim with an accompanying diagnosis of neoplasm (ICD9 diagnosis codes 140–239) in each Hospital Service Area (HSA). HSAs are local health care markets for hospital care. An HSA is a collection of ZIP codes whose residents receive most of their hospitalizations from the hospitals in that area [34]. There are 3,436 HSAs in the U.S. and 208 in Texas. The Dartmouth Atlas, which defined HRRs and HSAs, aggregates the HSAs to define HRRs. Specialist types were defined based on the treatment guideline being examined. For example, for surgical removal of lymph nodes during colorectal resection, we counted the number of surgeons per HSA. But for radiation therapy for a diagnosis of regional rectal cancer, the number of radiation oncologists was counted. Specialists associated with each cancer are listed in Additional file 1: Table S1.

### Statistical analysis

We first report the mean treatment rates for each cancer type in our study across all patients in Texas. We then report the treatment rate in the median HRR in the state for each recommended course of care, as well as the lowest and highest treatment rates by HRR. We list the name of the HRR associated with each reported rate, so that we can look for similarities and differences in cancer care across regions in Texas.

In regression analyses for each treatment, the dependent variable was set equal to 1 if the patient received the recommended treatment given their diagnosis and 0 otherwise. We first estimated logistic regressions that included only the HRRs as explanatory variables to test for variation in appropriate treatment across parts of Texas. For each treatment, we set the HRR with the median treatment rate for the sample as the excluded category in the regressions. We then tested for differences in treatment rates across HRRs, adjusting for patient characteristics, census tract-level characteristics, and the supply of cancer specialists in the HSA. Cancer stage (local, regional, or distant versus *in situ*) was included in the colon cancer regression. For the other treatments, cancer stage was used to define the cancdidate treatment population and was therefore not a regressor. We include the supply specialists when measuring the determinants of favorable treatment for prostate cancer, because we seek to test whether the overly high rate of cancer-directed treatment for this patient group may be an example of “supply-sensitive care.” This term refers to clinical activities for which the frequency of use is related to the capacity of the local healthcare system, but which do not yield better health outcomes [11, 35]. All regression analyses were conducted using Stata 11.2, and standard errors were computed using the cluster option to account for correlation in unobservables across HRRs [36]. Means of all of the explanatory variables included in the regressions are listed by treatment status in Additional file 1: Table S2.

## Results

Table 1 provides descriptive statistics on mean treatment rates for the 7 recommended courses of cancer care, as well as information on the Texas HRRs with median, minimum, and maximum treatment rates. We report the number of cancer patients eligible for each recommended treatment in each HRR in a table in the additional file. The mean and median treatment rates for each treatment are very close, suggesting that even if there are HRRs with outlier treatment rates, these rates are balanced above and below the median HRR.

**Table 1:** Mean treatment rates for recommended courses of care

	All stages colon cancer	Regional colon cancer	Regional rectal cancer	Regional rectal cancer with resection	Favorable non-metastatic prostate cancer	Unfavorable non-metastatic prostate cancer	Locoregional resectable pancreatic cancer
Treatment Rate %(n/N)	resection w/12+ nodes^a^	chemotherapy^b^	radiation therapy^c^	postoperative chemotherapy^d^	no treatment^e^	any treatment^f^	resection^g^
Mean of TX	48 %(2760/5809)	54 %(1089/2009)	61 %(407/668)	48 %(248/518)	21 %(1119/5220)	88 %(5240/5978)	41 %(275/673)
Median	Lubbock 48 %(77/161)	Austin 56 %(51/91)	Wichita Falls 60 %(6/10)	Austin 54 %(19/35)	Houston 21 %(223/1081)	McAllen 89 %(48/54)	Beaumont 40 %(8/20)
	95 % CI 41 % to 50 %	95 % CI 53 % to 60 %	95 % CI 58 % to 69 %	95 % CI 42 % to 59 %	95 % CI 18 % to 26 %	95 % CI 85 % to 91 %	95 % CI 36 % to 48 %
Minimum	Victoria 27 %(24/90)	Beaumont 44 %(21/48)	Temple 40 %(2/5)	El Paso 0 %(0/7)	Harlingen 8 %(5/60)	Amarillo 68 %(145/212)	San Antonio 22 %(15/67)
Maximum	Wichita Falls 59 % (51/86)	Longview 71 %(17/24)	Bryan 100 %(2/2)	Victoria 75 %(6/8)	Amarillo 36 %(53/149)	Odessa 96 %(48/50)	San Angelo 75 %(3/4)

*n* = total number of treated patient in each HRR, N = total number of patient in each HRR

^a^had colorectal resection with at least 12 nodes removed within 6 months of diagnosis

^b^had chemotherapy within 4 months of diagnosis

^c^had radiation therapy within 6 months of diagnosis

^d^had rectal resection within 6 months of diagnosis and postoperative chemotherapy

^e^did not have any of following treatments within 12 months of diagnosis: external beam radiation therapy, brachytherapy, prostatectomy, cryosurgery, or androgen deprivation therapy(orchiectomy or medical)

^f^had any of following treatments within 12 months of diagnosis: external beam radiation therapy, brachytherapy, prostatectomy, cryosurgery, or androgen deprivation therapy(orchiectomy or medical)

^g^had resection within 12 months of diagnosis

Similar to previous research, mean rates of recommended care for colorectal cancer patients in Texas are relatively low [28]. The rate of removal of 12+ lymph nodes during colon cancer resection is lowest in the HRR of Victoria (27 %) and highest in Wichita Falls (59 %). Differences between HRRs with the lowest and highest rates of chemotherapy for regional colon cancer are similarly wide (44 % for Beaumont, versus 71 % for Longview). We do not have sufficient numbers of patients with rectal cancer in all HRRs across Texas to draw conclusions regarding this patient group.

Similar to past research, few older men with a favorable prognosis for prostate cancer are managed with observation and instead receive cancer-directed treatment. Most men with unfavorable prostate cancer receive cancer-directed treatment such as prostatectomy or external beam radiation. Even so, treatment rates for prostate cancer patients differ markedly across HRRs. Amarillo has the highest rate of recommended treatment for patients with favorable risk (36 %), but the lowest rate of recommended treatment for patients with unfavorable risk profiles (68 %). Harlingen has the lowest rate of appropriate care for prostate cancer patients with favorable risk (8 %), and Odessa has the highest rate of treatment for patients with unfavorable risk (96 %).

The mean rate of resection for pancreatic cancer patients with locoregional disease is only 41 %, similar to findings from past research [15]. San Antonio has the lowest rate of surgical resection (22 %), while San Angelo has the highest rate (75 %). Given that the total number of patients diagnosed with resectable logoregional pancreatic cancer during the sample period is relatively low (673), and the numbers of patients in the sample with regional rectal cancer is also small (668), we test for significant differences across regions of Texas using regression analysis.

Table 2 contains results of logistic regressions where we test for differences in the probability of receiving recommended treatment across HRRs, without adjustment for other patient factors. We find 8 out of 22 HRRs in Texas have rates of lymph node removal with colon cancer surgery which are significantly different from the median region of Lubbock. The regressions reveal no significant differences in rates of chemotherapy for patients with regional colon cancer. We also find no significant difference across HRRs in either recommended therapies for rectal cancer patients.

**Table 2:** Unadjusted odds of receiving recommended treatment by hospital referral region

	All stages colon cancer with colorectal Resection 12+ nodes	Regional colon cancer with chemotherapy	Regional rectal cancer with radiation therapy	Regional rectal cancer with postoperative chemotherapy	Favorable non-metastatic prostate cancer without treatment	Unfavorable non-metastatic prostate cancer with any treatment	Locoregional resectable pancreatic cancer with resection
Treatment Rate	48 %	54 %	61 %	48 %	21 %	88 %	41 %
Hospital referral region							
Abilene	**0.6 (0.03)**	1.3	0.7	1.7	1.4	0.9	2.3
Amarillo	1.5	1.3	1.7	1.3	**2.1**	**0.3 (<0.01)**	1.7
Austin	**1.5 (0.05)**	1	0.8	1	**0.5 (<0.01)**	2.3	0.9
Beaumont	**0.6 (0.01)**	0.6	1.7	1.7	**1.8 (<0.01)**	0.7	1
Bryan	1.5	1.4	(omitted)	(omitted)	1.2	1.3	0.5
Corpus Christi	**0.6 (0.01)**	0.7	1.9	1.2	1.2	1.4	0.6
Dallas	1	0.9	1.2	0.6	**0.7 (<0.01)**	1.3	1.3
El Paso	1.2	0.7	2.2	(omitted)	1.4	0.7	0.8
Fort Worth	1.2	0.9	0.8	1.1	0.9	0.8	1.2
Harlingen	**0.6 (0.01)**	1.1	0.7	0.8	**0.4 (0.03)**	3	0.6
Houston	1.1	0.9	0.9	0.6	1	0.9	1.1
Longview	1.2	1.9	1	0.5	0.9	1.4	0.9
Lubbock	1	1.6	0.8	1.2	1	1.1	1.5
McAllen	**0.5 (<0.01)**	1.6	1.1	1	1.6	1	1.3
Odessa	1.2	0.8	1.5	1.5	0.8	3	3
San Angelo	0.8	0.6	2	2.1	**2 (<0.01)**	0.6	4.5
San Antonio	1.2	0.8	0.8	0.6	**1.7 (<0.01)**	0.4	0.4
Temple	0.6	1.2	0.4	1.7	1.5	0.7	2
Tyler	1.2	1.2	1.3	1	**1.5 (0.01)**	1	1
Victoria	**0.4 (<0.01)**	1.4	1	2.5	0.5	1.1	0.6
Waco	**0.6 (0.03)**	0.8	1	1	0.8	1	0.9
Wichita Falls	1.6	1.2	1	0.6	0.4	1.8	0.8
Sample Size	5809	2009	668	518	5220	5978	673

Numbers in parentheses are p-values. Bolded coefficients have p-values less than 0.05. For ease of reading, p-values greater than 0.05 are not reported

The regressions indicate that 3 HRRs have odds ratios of recommended care for favorable prostate cancer patients which are lower than the median HRR of Houston, where 21 % of patients are delivered cancer-directed treatment despite recommendations not to do so. Among prostate cancer patients with unfavorable risk profiles, we found only one HRR with a treatment rate that was significantly different from the median HRR of McAllen. We found no statistically significant differences in unadjusted resection rates for pancreatic cancer patients across HRRs.

**Fig. 1:**
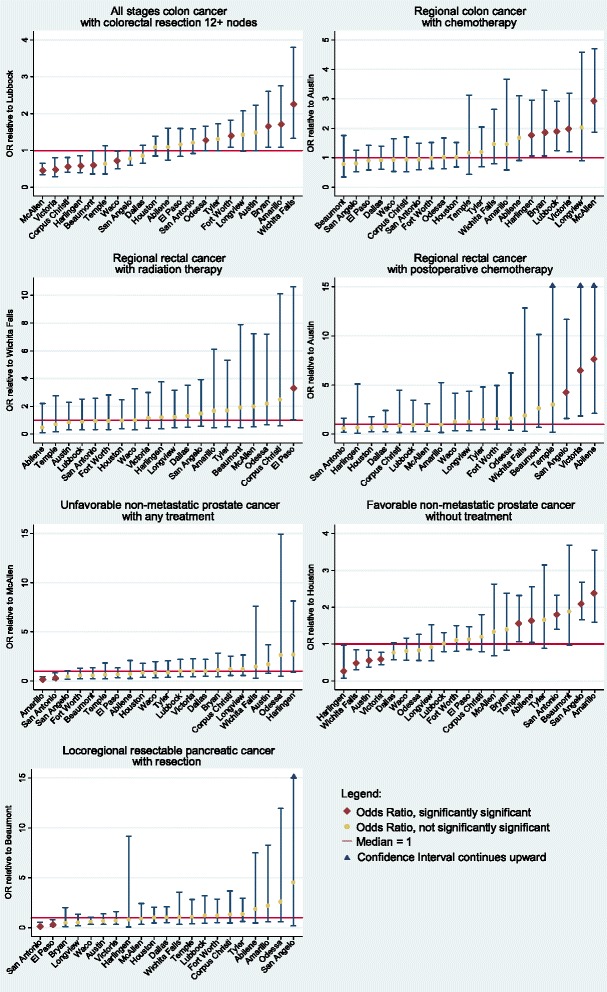
Adjusted Odds of Receiving Recommended Treatment by HRR

Table 3 reports results of logistic regressions testing for significant differences in treatment rates across HRRs, adjusted for patient characteristics and local specialist supply. These odds ratios are also graphed in Fig. 1, with statistically significant odds ratios in red and statistically insignificant odds ratios in yellow. Descriptive statistics on the means for each of the explanatory variables in the regressions are listed in a table in the additional file. The number of HRRs with rates of removal and examination of lymph nodes for resected colon cancer patients rises to 13, versus 8 in the unadjusted results. A total of 7 HRRs have removal rates which are significantly lower than the median HRR of Lubbock. In McAllen, the lowest outlier, the odds ratio for removal of 12+ lymph nodes for colon resection patients is 0.5 (*p* < 0.01). The highest outlier, Wichita Falls, has an odds ratio of removal of 12+ lymph nodes equal to 2.3 (*p* < =0.01).

**Table 3:** Adjusted odds of receiving recommended treatment by hospital referral region

	All stages colon cancer with colorectal Resection 12+ nodes	Regional colon cancer with chemotherapy	Regional rectal cancer with radiation therapy	Regional rectal cancer with postoperative chemotherapy	Favorable non-metastatic prostate cancer without treatment	Unfavorable non-metastatic prostate cancer with any treatment	Locoregional resectable pancreatic cancer with resection
Treatment Rate	48 %	54 %	61 %	48 %	21 %	88 %	41 %
Hospital referral region							
Abilene	1.1	1.7	0.5	**7.7 (<0.01)**	**1.6 (0.03)**	0.71	1.9
Amarillo	**1.7 (0.02)**	1.5	1.7	1	**2.4 (<0.01)**	**0.2 (<0.01)**	2.2
Austin	1.5	1	0.8	1	**0.6 (0.01)**	1.7	0.7
Beaumont	**0.6 (0.05)**	0.8	1.9	2.6	1.9	0.6	1
Bryan	**1.7 (0.02)**	**1.9 (0.03)**	(omitted)	(omitted)	1.4	1.1	0.5
Corpus Christi	**0.6 (<0.01)**	1	2.5	0.8	1.2	1.2	1.3
Dallas	0.9	0.9	1.3	0.8	0.8	1	1
El Paso	1.2	0.9	**3.3 (0.04)**	(omitted)	1.1	0.7	**0.4 (0.02)**
Fort Worth	**1.4 (0.01)**	1	1	1.6	1.1	0.5	1.2
Harlingen	**0.6 (<0.01)**	**1.8 (0.03)**	1.2	0.7	**0.3 (0.04)**	2.7	0.8
Houston	1.1	1	1	0.7	1	0.8	1
Longview	1.4	2	1.2	1.3	0.9	1.2	0.5
Lubbock	1	**1.9 (<0.01)**	0.9	1	1	1	1.2
McAllen	**0.5 (<0.01)**	**3.0 (<0.01)**	2	0.9	1.3	1	0.9
Odessa	**1.3 (0.05)**	1	2.2	1.6	0.8	2.6	2.6
San Angelo	0.8	0.8	1.5	**4.3 (<0.01)**	**2.1 (<0.01)**	0.4	4.5
San Antonio	1.2	1	0.9	0.6	**1.8 (<0.01)**	**0.3 (0.01)**	**0.3 (<0.01)**
Temple	0.6	1.2	0.7	3	**1.6 (0.02)**	0.6	1.1
Tyler	1.3	1.2	1.7	1.4	1.7	0.9	1.4
Victoria	**0.5 (<0.01)**	**2.0 (<0.01)**	1.1	**6.57 (<0.01)**	**0.6 (<0.01)**	1	0.7
Waco	**0.7 (0.04)**	0.9	1	1.2	0.8	0.8	0.6
Wichita Falls	**2.3 (<0.01)**	1.5	1	1.9	**0.5 (0.01)**	1.5	1.1
Sample Size	5809	2009	668	518	5220	5978	673

Numbers in parentheses are p-values. Bolded coefficients have p-values less than 0.05. For ease of reading, p-values greater than 0.05 are not reported

Unlike the unadjusted results, five HRRs have odds ratios of chemotherapy for patients with regional colon cancer which are significantly different (and higher) compared to the median HRR of Austin. McAllen is the highest outlier, with an odds ratio of chemotherapy equal to 3.0 (*p* < 0.00).

Among rectal cancer patients, there were no significant differences in treatment rates across HRRs in the unadjusted results. In the adjusted results, there were four HRRs with significantly different treatment rates relative to the median HRR. In El Paso, the odds ratio of radiation therapy for patients with regional rectal cancer was 3.3 (*p* = 0.04) relative to Wichita Falls. Abilene, San Angelo and Victoria all had significantly higher odds ratios of postoperative chemotherapy for patients with regional rectal cancer (7.7, *p* < 0.01; 4.3, *p* < 0.01; and 6.6, *p* < 0.01 respectively).

The adjusted regressions show nine HRRs have treatment rates for prostate cancer patients with favorable risk to be significantly different from the median (as opposed to only 3 HRRs in the unadjusted results). The lowest outlier, Harlingen, has an odds ratio equal to 0.3 (*p* = 0.04) relative to the median HRR of Houston. The highest outlier is Amarillo, with an odds ratio equal to 2.4 (*p* < 0.01). Among prostate cancer patients with unfavorable risk, only two HRRs had treatment rates that were significantly different from the median rate in McAllen. Amarillo is again the lowest outlier, with an odds ratio of 0.20 (*p* < 0.01). Unlike the unadjusted comparisons, two HRRs had rates of pancreatic resection that were significantly lower than the median HRR (Beaumont) in the adjusted regressions. El Paso had an odds ratio of 0. (*p* = 0.0), and San Antonio had an odds ratio of 0.3 (*p* < 0.01).

In the adjusted regressions, the patient characteristics gender, age category, race/ethnic origin, rural/urban location, Charlson score, tumor size, year of diagnosis, and median income are each significantly associated with the probability of receiving treatment for at least one recommended cancer treatment type. Older age, being black, having two or more Charlson comorbidities, and living in a lower income area all reduce the likelihood of receiving treatment for at least three recommended therapies. The year of diagnosis affects the likelihood of treatment in 4 of the 7 treatment types, but not in a systematic manner. The presence of a teaching hospital in an HRR does not appear to systematically influence treatment rates. Dallas and Houston have the highest number of teaching hospitals, but neither area has significantly higher or lower treatment rates than the median HRR. In contrast, Amarillo, which houses a teaching hospital for Texas Tech, reports a significantly higher rate of colon cancer resections with 12 or more lymph nodes removed; and a significantly lower rate of prostate cancer patients with an unfavorable prognosis receiving treatment. We could not formally test for the association between the number of teaching hospitals and treatment rates in this sample. Only 7 of 22 HRRs had 1 or more teaching hospitals, and 4 had only one present. Therefore there was not enough variation in the number of teaching hospitals to overcome multicollinearity issues.

The local supply of specialists in the area where each patient is treated is not an important determinant of whether a patient receives recommended care. Colorectal cancer patients who were resected in HSAs with 10 or more surgeons per cancer patient were more likely to have 12+ lymph nodes removed with surgery (OR = 1.61, *p* < 0.001), but such effects were not precisely estimated for any of the other recommended cancer therapies. Neither the number of radiation oncologists or urologists per cancer patient were significant predictors of the probability of treatment for patients with a favorable prognosis for prostate cancer treatment. Therefore, the theory supply sensitive care does not appear to explain the tendency to practice aggressive treatment for this population instead of active surveillance.

## Discussion

Previous studies have already identified low rates of appropriate treatment in the U.S. for the cancers that we consider [15, 16, 28, 37]. For example, a previous analysis of SEER data found that only 37 % of 116,995 colorectal cancer patients received adequate lymph node evaluation [38]. This paper tests for regional variation in treatment rates around these low absolute levels of appropriate care. We also examine multiple cancer types simultaneously, to determine whether there are regions which tend to be more or less aggressive in treatment.

We find at least some significant difference in treatment rates for 7 recommended therapies across regions of Texas. The variation in treatment rates are particularly notable for colon cancer and for prostate cancer patients with favorable risk. Of 22 HRRs in Texas, the likelihood that 12+ lymph nodes are removed for resected colon cancer patients is significantly higher or lower for 13 HRRs relative to the median HRR.

These differences are not due to variation in patient characteristics across regions. When we adjust for patient characteristics, the disparities are actually wider. Among prostate cancer patients with a favorable risk profile, 9 HRRs have treatment rates significantly higher or lower than the median region. The differences also do not appear to be associated with population size. Most of the smaller HRRs [39] have significantly higher or lower rates of treatment relative to the median for at least one recommended cancer treatment. However, San Antonio is the third largest HRR in Texas in terms of Medicare beneficiaries, and it has significantly different treatment rates for both prostate cancer treatments and pancreatic resection. In addition, Fort Worth and Austin, the fourth and fifth largest HRRs, have significantly higher rates of lymph node resection for patients with colorectal cancer.

Some past literature concludes that certain regions provide more aggressive health care than others [11, 40]. However, these studies examined aggregate measures of health care utilization, such as the number of medical specialist visits or the percent of hospital patients admitted to an intensive care unit in a region. In contrast, we find that regions can provide above or below median rates of treatment when one looks at multiple different cancer types and recommended treatments. No HRR performs systematically better or worse in treatment levels for all 7 recommended courses of cancer care that we studied. For each individual HRR, one can find odds ratios of receiving recommended treatment that are greater or less than one across the 7 courses of care that we examine. The HRR with the median rate of treatment is different for each of the 7 therapies as well.

Multiple studies associate the availability of primary care physicians and the role they play in screening with improved cancer survival [19–26]. One recent study found an association between increased specialist supply and lower cancer mortality across counties in the U.S. [25]. However, we found only one instance out of several (availability of surgeons for removal of lymph nodes for colorectal cancer resection) in which the availability of specialists is associated with receipt of appropriate care. With the large increase in insured persons following the implementation of the Affordable Care Act, some policy makers have been concerned that greater demand for health care will lead to a physician shortage. Patients may face difficulty obtaining screening and referrals from primary care physicians, but increased demand for specialist care may not be the most important factor in determining delivery of appropriate treatment for cancer patients. Insurance coverage is also an important determinant for whether a patient receives treatment, but our analyses was based on patients covered by Medicare.

It may be that the underlying factors influencing differing rates of treatment across regions vary by cancer type. Different specialists are responsible for treating the three major cancers we consider, and their practice styles and referral patterns may be an important determinant of variations in care. These differences do not appear to be influenced by proximity to teaching hospitals, because treatment rates are either higher or lower than the median in each HRR, regardless of the presence of teaching hospitals in each region.

Our study has certain caveats. The mean rate of treatment for colorectal and pancreatic cancer patients is so low, that there may be “more room” for variation among HRRs than one might observe elsewhere in the U.S. However, the rate of pancreatic resection of 34 % measured in our study is comparable to the 28.6 % rate of surgical resection identified in a previous nationwide study [15]. The absence of regional variation in recommended treatments for rectal cancer may be due to the relatively small number of patients with this cancer in our sample. We also lack information that would allow us to determine how much low levels of treatment are attributable to physician actions versus patient refusal to accept treatment, or financial barriers to receiving care.

For the three of the seven recommended treatments, there were only one or two regions out of 22 that had significantly different adjusted treatment rates from the median HRR. It is possible that one or two regions appeared significantly different for these treatments due to random chance, given that so many regions were simultaneously compared to each other. Unfortunately, we do not have an appropriate method for adjusting the p-value below 0.05 to account for these multiple comparisons. A Bonferroni correction can be applied to adjust p-values when one multiple independent tests of the same hypothesis. However, the regional comparisons in this analysis are correlated rather than independent [41].

Because we are analyzing Medicare claims data, we are missing information on patients under age 65 in the U.S. Physicians are more likely to treat patients under age 65 more aggressively, [42] although regional variation in the propensity of cancer patients to receive treatment may persist for this younger population. The majority of patients under age 65 have privately purchased health insurance. However, Texas has the highest rate of uninsured adults ages 18 to 64 in the U.S., with as many as 25 % uninsured during the study period [43]. Lack of health insurance has been associated with diagnosis at a later stage and shorter survival time [44]. Because U.S. residents are covered by multiple private health insurance companies, it is prohibitively expensive to collect data for the under age 65 population that is as comprehensive as the TCR-Medicare data used in this study.

## Conclusion

Our results suggest that regions in Texas differ widely in adherence to recommended treatment for seven different cancer interventions. These differences are not due to the availability of specialists or the presence of teaching hospitals. The absence of consistent explanators suggests that variations like these are likely to occur elsewhere nationwide. Regions with high rates of success in one treating one type of cancer cannot be assumed to excel in other areas. Moreover, previous studies that classify local areas as high-use or low-use may mask important differences within areas with respect to adherence to treatment guidelines.

Identifying the underlying causes of regional differences in adherence to guidelines will require more in-depth studies than have so far been conducted. A comprehensive review of the literature on barriers to guideline adherence identified multiple barriers to adherence. However, most studies only examined one or two potential causes [45]. The review divided studies into barriers due to physician knowledge, attitudes, and behavior. Examples that may be particularly relevant to cancer treatment include lack of familiarity with guidelines, lack of agreement in the benefits of treatment versus the risks, inertia of previous practice, and patient-related barriers. Future studies of adherence to recommended treatment should be designed to examine all of these possibilities.

Other researchers suggest that more emphasis should be placed on Continuing Medical Education programming that disseminates recent guideline changes to physicians. With the dissemination and improvement in electronic health records, more quality monitoring at the physician level could help to raise overall levels of adherence for all HRRs [46]. A particularly promising model for Texas may be Michigan’s Oncology Quality Consortium [47]. This group represents a consortium of 40 physician organizations from across Michigan who received funding from Blue Cross Blue Shield of Michigan to collect patient data to track their quality of care. The information has allowed the organization to design quality and process improvement interventions. Physicians may have more success obtaining financial support from private insurers to improve cancer care at the state level, because insurers can attract more customers if they can demonstrate higher quality care in their network of providers. Adherence to guidelines may also lower the costs of cancer care for insurers [48, 49].

## Additional files

Additional file 1: Table S1. Specialties* Treating Cancer Patients. Types of medical specialists treating specific cancers. **Table S2.** Number of Cancer Patients Eligible for Care by HRR and Treatment Type. Number of cancer patients in Hospital Referral Regions eligible for care by treatment type. **Table S3.** Descriptive statistics^a^ for the treatment vs. non-treatment group. Statistics for treated vs. non-treated for selected variables. (DOCX 28 kb)
