# Supplementary material for: Regional differences in recommended cancer treatment for the elderly
Source: BMC Health Serv Res. 2016 Jul 15;16:262. doi: 10.1186/s12913-016-1534-z (PMC4946160; doi:10.1186/s12913-016-1534-z)
Supplement: Additional file 1: Table S1. — Specialties* Treating Cancer Patients. Types of medical specialists treating specific cancers. Table S2. Number of Cancer Patients Eligible for Care by HRR and Treatment Type. Number of cancer patients in Hospital Referral Regions eligible for care by treatment type. Table S3. Descriptive statisticsa for the treatment vs. non-treatment group. Statistics for treated vs. non-treated for selected variables. (DOCX 28 kb) [file 12913_2016_1534_MOESM1_ESM.docx]

**Additional files**

**Regional differences in recommended cancer treatment for the elderly**

| **Table S1:** | |
| --- | --- |
| **Specialties* Treating Cancer Patients** | |
|  |  |
| **Type of Cancer** | **Specialty** |
| **Colon Cancer** |  |
|  | Medical Oncology |
|  | Surgery |
|  | General Surgery |
|  | Colon and Rectal Surgery |
|  | Surgical Oncology |
|  |  |
| **Rectal Cancer** |  |
|  | Medical Oncology |
|  | Hematology |
|  | Hematology/Oncology |
|  | Radiation Oncology |
|  |  |
| **Pancreatic Cancer** |  |
|  | Surgery |
|  | General Surgery |
|  | Surgical Oncology |
|  |  |
| **Prostate Cancer** |  |
|  | Radiation Oncology |
|  | Urology |

*Specialties were obtained from the American Medical Association Physician Masterfiles.

| **Table S2: Number of Cancer Patients Eligible for Care by HRR and Treatment Type** | | | | | | | |
| --- | --- | --- | --- | --- | --- | --- | --- |
|  | **All stages colon cancer** | **Regional colon cancer** | **Regional rectal cancer** | **Regional rectal cancer with resection** | **Favorable non –metastatic prostate cancer** | **Unfavorable non-metastatic prostate cancer** | **Locoregional resectable pancreatic cancer** |
| Treatment rate  (n/N)% | Resection w/12+ nodes | chemotherapy | Radiation therapy | Postoperative chemotherapy | No treatment | Any treatment | Resection |
| Abilene | 185 | 59 | 6 | 6 | 108 | 78 | 10 |
| Amarillo | 161 | 54 | 14 | 10 | 149 | 212 | 17 |
| Austin | 276 | 91 | 42 | 35 | 376 | 488 | 38 |
| Beaumont | 186 | 48 | 25 | 24 | 185 | 135 | 20 |
| Bryan | 77 | 28 | 2 | 1 | 80 | 88 | 8 |
| Corpus Christi | 153 | 50 | 19 | 12 | 69 | 180 | 10 |
| Dallas | 962 | 351 | 115 | 95 | 785 | 858 | 129 |
| El Paso | 162 | 60 | 13 | 7 | 245 | 285 | 21 |
| Fort Worth | 426 | 144 | 45 | 36 | 526 | 319 | 69 |
| Harlingen | 126 | 43 | 8 | 8 | 60 | 100 | 22 |
| Houston | 1353 | 487 | 161 | 115 | 1081 | 1552 | 122 |
| Longview | 91 | 24 | 15 | 14 | 60 | 122 | 8 |
| Lubbock | 161 | 49 | 17 | 12 | 177 | 213 | 22 |
| McAllen | 131 | 55 | 16 | 13 | 59 | 54 | 15 |
| Odessa | 145 | 51 | 16 | 14 | 84 | 50 | 9 |
| San Angelo | 59 | 16 | 12 | 7 | 93 | 56 | 4 |
| San Antonio | 499 | 207 | 70 | 52 | 493 | 530 | 67 |
| Temple | 74 | 20 | 5 | 3 | 65 | 83 | 7 |
| Tyler | 277 | 80 | 24 | 22 | 318 | 339 | 44 |
| Victoria | 90 | 17 | 10 | 8 | 52 | 97 | 11 |
| Waco | 129 | 42 | 23 | 17 | 103 | 92 | 11 |
| Wichita Falls | 86 | 33 | 10 | 7 | 52 | 47 | 9 |

| **Table S3: Descriptive statistics^a^ for the treatment vs. non-treatment group** | | | | | | | |
| --- | --- | --- | --- | --- | --- | --- | --- |
|  | **All stages colon  cancer** | **Regional colon  cancer** | **Regional rectal  cancer** | **Regional rectal cancer with resection** | **Favorable  non-metastatic  prostate cancer** | **Unfavorable  non-metastatic  prostate cancer** | **Locoregional  resectable  pancratic cancer** |
| **Treatment and Treatment rate** | **resection w/12+ nodes** | **chemotherapy** | **radiation therapy** | **postoperative  chemotherapy** | **no treatment** | **any treatment** | **resection** |
|  | 48% | 54% | 61% | 48% | 21% | 88% | 41% |
| **Age at diagnosis  treated  not treated** | 73.0  72.9 | 72.3 74.0 | 71.9 73.0 | 72.2 72.6 | 72.0 74.2 | 72.9 75.3 | 74.2 77.7 |
| **Charlson score  treated  not treated** | 0.8 0.9 | 0.6 1.0 | 0.5 0.7 | 0.5 0.7 | 0.4 0.7 | 0.5 0.6 | 0.8 1.1 |
| **Tumor size in mm  treated  not treated** | 166.2 391.2 | 146.8 169.4 | 293.8 171.6 | 125.1 295.9 | 913.9 925.1 | 884.7 904.6 | 100.9 354.7 |
| **Median income  treated  not treated** | $41037 $38636 | $40380 $39533 | $39981 $40044 | $40057 $40185 | $43377 $40070 | $42653 $39818 | $42382 $41355 |
| **Some college  treated  not treated** | 27.6 27.2 | 27.3 26.7 | 27.1 27.5 | 26.9 27.6 | 28.2 28.1 | 27.7 27.7 | 27.6 27.4 |
| **% no English  treated  not treated** | 6.0 6.5 | 6.7 7.0 | 6.12 7.0 | 6.2 6.6 | 5.6 6.5 | 6.2 6.9 | 5.9 6.8 |
| **Urban residence  treated %  not treated %** | 84.2 82.2 | 82.3 84.6 | 79.4 86.6 | 80.2 84.4 | 83.7 81.1 | 83.3 83.9 | 85.8 86.7 |
| **Male   treated %  not treated %** | 46.2 52.2 | 49.5 46.5 | 61.4 62.8 | 62.1 63.0 | 100 100 | 100 100 | 48.4 43.2 |
| **Race: Black   treated %  not treated %** | 8.8 11.5 | 8.5 9.9 | 7.9 10.0 | 4.8 9.3 | 7.7 11.7 | 8.2 14.8 | 7.3 8.3 |
| **Hispanic   treated %  not treated %** | 13.2 14.6 | 15.9 15.2 | 15.5 16.5 | 16.5 17.4 | 11.1 13.0 | 12.3 12.3 | 14.9 16.8 |
| **White   treated %  not treated %** | 74.6 69.9 | 72.8 71.2 | 74.2 70.9 | 76.2 70.7 | 77.1 71.1 | 75.4 69.3 | 76.4 72.1 |
| **Other   treated %  not treated %** | 3.4 4.0 | 2.8 3.7 | 2.5 2.7 | 2.4 2.6 | 4.2 4.3 | 4.1 3.7 | 1.5 2.8 |
| **Year of diagnosis:  2004   treated %  not treated %** | 23.3 31.3 | 29.7 26.9 | 33.7 29.2 | 39.5 26.7 | 28.4 26.0 | 23.3 24.5 | 20.4 25.4 |
| **2005   treated %  not treated %** | 23.8 27.8    27.80 27.80 | 25.0 24.4 | 23.1 23.8 | 21.8 20.4 | 25.7 27.1 | 24.0 23.6 | 30.18 24.37 |
| **2006  treated %  not treated %** | 25.3 22.1 | 22.6 26.0 | 24.3 21.5 | 21.4 27.8 | 24.4 21.5 | 26.0 22.4 | 22.9 22.9 |
| **2007  treated %  not treated %** | 27.6 18.9 | 22.8 22.8 | 18.9 25.7 | 17.4 25.2 | 21.5 25.4 | 26.7 29.5 | 26.6 27.4 |
| **Stage: In situ   treated %  not treated %** | 1.8 6.9 |  |  |  |  |  |  |
| **Local   treated %  not treated %** | 39.0 39.4 |  |  |  |  |  |  |
| **Regional   treated %  not treated %** | 45.1 25.0 |  |  |  |  |  |  |
| **Distant  treated %  not treated %** | 13.6 20.7 |  |  |  |  |  |  |
| **Surgeons per  1000 cancer patients*** | 6.3 |  |  |  |  | | 6.3 |
| **Oncologists per 1000 cancer patients*** |  | 3.7 |  | 3.2 |  | |  |
| **Urologists per  1000 cancer patients*** |  |  |  |  | 2.9 | |  |
| **Rad. Oncologists per 1000 cancer patients*** |  |  | 1.1 |  | 1.2 | |  |
| ^a^ Values represent means unless otherwise indicated. | | | | | | | |
| * The denominator is the number of Medicare patients in the HSA with a cancer diagnosis. | | | | | | | |
